# Expression of IL-5 receptor alpha by murine and human lung neutrophils

**DOI:** 10.1371/journal.pone.0221113

**Published:** 2019-08-15

**Authors:** Stacey A. Gorski, Monica G. Lawrence, Amy Hinkelman, MarthaJoy M. Spano, John W. Steinke, Larry Borish, W. Gerald Teague, Thomas J. Braciale

**Affiliations:** 1 Carter Center for Immunology Research, University of Virginia, Charlottesville, VA, United States of America; 2 Department of Medicine, Division of Asthma, Allergy and Immunology, University of Virginia, Charlottesville, VA, United States of America; 3 Department of Pediatrics, Division of Pediatric Respiratory Medicine, Allergy and Immunology, University of Virginia, Charlottesville, VA, United States of America; 4 Department of Pathology, University of Virginia, Charlottesville, VA, United States of America; Louisiana State University System, UNITED STATES

## Abstract

The role of eosinophilia in atopic diseases, including asthma, is well established, as is the well-known role of IL-5 as a major eosinophilopoeitin and chemoattractant. Following influenza A virus infection of mice, type 2 innate lymphoid cells are recruited to the respiratory tract and produce large quantities of IL-5, which contributes to the recruitment of eosinophils into the infected lungs during the recovery phase of infection. We demonstrate here that while IL-5 is required for optimal recovery from influenza A virus infection in BALB/c and C57BL/6 mice, the protective effect of IL-5 is independent of eosinophils, suggesting an alternative cellular target. We describe the unexpected finding of IL-5 receptor alpha (CD125) expression on neutrophils infiltrating the inflamed mouse lungs, as well as on neutrophils at other anatomic sites. We extend this finding of neutrophil CD125 expression to humans, specifically to neutrophils found in the bronchoalveolar lavage fluid from the inflamed lungs of children with treatment-refractory asthma. We further demonstrate that the IL-5 receptor on neutrophils is capable of signal transduction. Our data provide further evidence that neutrophils can play a role bridging atopic type 2 and innate anti-microbial immunity.

## Introduction

IL-5 has been extensively studied in the context of allergic disease and asthma, due to its critical role in regulating eosinophil biology. Eosinophils, as well as murine B-1 cells, require IL-5 for proliferation, differentiation, and egress out of the bone marrow and into the circulation [1–6]. The IL-5 receptor (IL-5R) is a heterodimer of the cytokine-binding alpha (α) chain (CD125) and the signal-transducing common beta (β) chain (CD131), the latter chain of which is also shared by the receptors for granulocyte-macrophage colony-stimulating factor (GM-CSF) and IL-3 [7]. IL-5R is classically thought to be expressed on eosinophils and basophils, but has also been reported to be expressed by activated B cells, airway epithelium activated by recombinant IL-5 and, in conflicting reports, group 2 innate lymphoid cells (ILC2) [8–10]. Engagement of the IL-5R on eosinophils primarily results in transient phosphorylation of STAT5, with a possible minor contribution from STAT1 dependent signaling [11–13].

Airway epithelial damage by allergen and/or infection leads to the production of TSLP, IL-25 and IL-33 [14, 15], which in turn stimulate ILC2 cells to produce large quantities of IL-5 and IL-13. We have previously shown that ILC2 cells are recruited to the respiratory tract of mice following influenza A virus (IAV) infection and produce large quantities of IL-5 beginning 4-days post infection (d.p.i). There is subsequent ILC2-mediated recruitment of eosinophils into the infected lungs during the recovery phase of infection (8–16 d.p.i.) [16]. Previous studies have demonstrated the recruitment of eosinophils following IAV infection [17–19], and emerging evidence supports the importance of eosinophils in anti-viral immunity to IAV, potentially via interaction with CD8+ T cells [20, 21]. Interestingly, during IAV infection, IL-5 released into bronchoalveolar lavage fluid (BALF) peaks at 6–8 d.p.i. and is no longer detected in BALF following infectious viral clearance [16]. However, abundant IL-5 gene mRNA remains readily demonstrable in lung homogenates well beyond this time. This raises the possibility of capture (and consumption) of IL-5 by IL-5R expressing cells that infiltrate the lungs during the recovery phase of infection starting at 8 d.p.i.

Here, we demonstrate that IL-5 is required for optimal recovery from IAV infection in mice, and that the protective effect of IL-5 is independent of eosinophils, suggesting an alternative cellular target. We describe the unexpected finding that following infection IL-5R is also expressed on lung-infiltrating neutrophils. We further demonstrate that these neutrophils are responsive to IL-5 signaling, and that a downstream effect of this signaling is suppression of reactive oxygen species (ROS) formation. We extended this finding of IL-5R expression to humans, specifically to neutrophils found in BALF and blood from children with treatment-refractory asthma and other inflammatory lung conditions.

## Materials and methods

### Mice

All animal experiments conducted in this study were carried out in accordance with the Animal Welfare Act and the recommendations in the Guide for the Care and Use of Laboratory Animals of the National Institutes of Health. All experiments were approved by the University of Virginia (UVA) Animal Care and Use Committee (ACAUC) (# 2230). BALB/c and C57BL/6 mice were purchased from the National Cancer Institute and PHIL mice were a kind gift from Dr. J. Lee and Dr. Nancy Lee (Mayo Clinic, Arizona) [22]. All mice used in experiments were between the ages of 8–12 weeks and matched for age and sex. All mice are initially housed in a pathogen-free “clean room” in individually ventilated cages with ¼ inch corncob bedding and free access to food (Envigo Tekland mouse/rat diet) and water with regulated light and dark cycles. The mice to be infected with influenza A are removed from the clean room to a satellite room where they are infected. They are housed in this infection room in the same way they are housed in the clean room; however, the cages of infected mice are placed in a large hood system with HEPA filtration. Their access to food and water is unchanged and they are monitored multiple times a day. They do not leave the infection room until the end of the experiment. Mice are sacrificed by cervical dislocation.

### Influenza A virus (IAV) infection

Type A influenza virus A/PR/8/34 (H1N1) was grown in day 10 chicken embryo allantoic cavities as described previously [23]. Mice were intra-nasally (i.n.) infected with 300 egg infectious doses (EID_50_) of A/PR/8/34 (corresponding to a 0.1 LD_50_ dose) unless otherwise stated.

### *In vivo* antibody administration

Mice were treated with 100 μg of IL-5 neutralizing antibody (Bio X Cell; clone TRFK5) i.p. daily, beginning at 7 d.p.i.

### Preparation of lung tissue

Single cell suspensions were prepared from lungs by mincing and digesting with dispase or collagenase followed by red blood cell lysis with ACK buffer, as described in detail previously [16].

### Analysis of murine BALF

As described in detail previously [16], BALF was obtained by cannulating the trachea and flushing the lungs with 0.5 ml of sterile PBS three times. Cells were removed by centrifugation and supernatants were stored at -80°C until analyzed. IL-5 levels in BALF were quantified by either ELISA (eBioscience, San Diego CA) or a multiplex Luminex assay (UVA Flow Cytometry Core Facility, Charlottesville VA). BAL fluid viral titers were determined via an endpoint dilution assay on Madin-Darby canine kidney cells (MDCK; American Type Culture Collection, Manassas VA) and expressed as tissue culture infectious dose_50_ (TCID_50_). MDCK cells were incubated with 10-fold dilutions of BAL fluid for 4 days in serum-free DMEM culture at 37°C with 5% CO_2_. Supernatants were collected and added at 1:1 to a solution of 1.5% chicken red blood cells (Charles River Laboratories, Wilmington MA). Hemagglutination was determined one half hour later.

### Analysis of murine serum

Mice were quickly opened and the hepatic portal vein was cut and 200–300 ul of blood was collected and placed in an EDTA tube. The blood was centrifuged at 2000 x g for 20 min to remove blood cells. The serum was stored at -80^°^C until ready to use. About 60–100 ul of serum was obtained per mouse. This serum was diluted 1:3 (55 ul of serum + 110 ul of assay diluent) so that cytokine levels could be measured in triplicate (60 ul of sample per well) via multiple Luminex assay (Flow Cytometry Core Facility at the University of Virginia).

### Flow cytometry and antibodies

BALF and blood were processed immediately after collection and kept on ice throughout. After red blood cell lysis, cells were suspended in FACS buffer containing PBS, 2% FBS, 10 mM EDTA and 0.01% NaN_3_. Suspensions were incubated with Fc receptor blocking antibody (eBioscience) to prevent non-specific binding. Cells were incubated on ice for 30 mins with a cocktail of antibodies to distinguish neutrophils from eosinophils. In mice, eosinophils were defined as CD45^+^CD11b^+^SiglecF^+^CD11c^lo^SSC^hi^ and neutrophils defined as CD45^+^CD11b^+^SiglecF^-^CD11c^+/-^Gr-1(Ly6G)^hi^. Sample gating strategy is shown in S1 Fig. The following mouse antibodies were used: CD11c, CD11b, CD45 (Biolegend); Gr-1(Ly6G), SiglecF (BD Biosciences, San Jose CA), CD125 (R&D Systems). Channels with significant auto-fluorescence were avoided. After acquisition on a FACS Canto II, data analysis was performed using FlowJo 9.8.5 (BD, Ashland OR). Fluorescence minus one (FMO) controls were used to define gates. Data are expressed as percentage of CD125+ neutrophils to allow for longitudinal analysis of data collected at different timepoints, and as geometric mean fluorescence intensity (gMFI) for within-experiment comparisons.

### HL-60 cell differentiation

The HL-60 cell line is derived from human promyelocytic leukemia and can be differentiated into neutrophil-like cells upon incubation with DMSO [24]. HL-60 cells were kept in RPMI 1640 media (supplemented with 10% FCS) with 1.5% DMSO (Sigma Aldrich, St. Louis MO) for the indicated number of days.

### Quantitative RT-PCR analysis

Total RNA was isolated from FACS sorted cell populations frozen in TRIzol according to the manufacturer’s instructions. RNA samples were treated with DNase I and complimentary DNAs (cDNA) were synthesized using random primers and Superscript II (all Invitrogen). Real time PCR of cDNA was performed in a StepOnePlus PCR system using SYBR Green master mix (Applied Biosystems, Foster City CA).

### pSTAT analysis

HL-60 differentiated cells were stimulated with rmIL-5 for 3, 5 or 10 min and were analyzed for pSTAT5 and/or pSTAT1 (Cell Signaling Technology, Danvers MA) via intracellular staining. Cells were fixed with BD Phosflow lyse/fix buffer followed by permeabilization with Phosflow Perm Buffer III (BD Biosciences). GM-CSF and IFN-γ were used as positive controls for pSTAT5 and pSTAT1, respectively.

### Reactive oxygen species (ROS) production

Whole lung cell suspensions were kept in culture for 1 hour prior to the addition of 10 μM 2′,7′-dichlorodihydrofluorescein diacetate (DCFA-DA; Sigma Aldrich). Cells were maintained at 37°C for 30 minutes prior to staining for surface markers for 10 minutes on ice and were then analyzed via flow cytometry for the presence of DCF generated via oxidation by ROS.

### Human subjects

Briefly, children (n = 16) were referred from the Central Virginia region to an academic specialty clinic for evaluation and treatment of poorly-controlled respiratory symptoms [25]. Children in whom all remediable/comorbid conditions were addressed, but whose symptoms remained poorly-controlled despite confirmation of adherence to treatment, were offered a diagnostic bronchoscopy under general anesthesia with BALF and blood samples shared between the clinical and research laboratories through protocols approved by the UVA Human Subjects Research Institutional Review Board (HSR-IRB) #17555, #10905, and #10634). Informed consent was provided by parents or legal guardians, and older children provided assent to participate in the protocol. Final diagnosis was based on results of the bronchoscopy, laboratory testing for atopy and presence of peripheral blood eosinophilia, presence of comorbid conditions such as eczema and food allergy, and response to treatment based on longitudinal follow-up. Flow analyses of BALF were performed as with the murine samples. The following human antibodies were used: CD45, Siglec8, CD66b (Biolegend, San Diego CA); CD125 Clone 26815 (R&D Systems, Minneapolis MN). In humans, eosinophils were defined as side scatter (SSC)^high^/CD45^+^/ CD66b^+^/Siglec 8^+^, and neutrophils were defined as SSC^high^/ CD45^+^/CD66b^+^/Siglec 8^-^).

### Statistical analysis

Statistical analyses were performed using Prism 7 (GraphPad Software, La Jolla CA). Unpaired, two-tailed Student t-test; one-way ANOVA; and paired, two-tailed t tests or Wilcoxon matched-pairs signed rank tests were used to determine significance (individual tests are labeled in figure legends). P values <0.05 were considered statistically significant.

## Results

### IL-5 is required for optimal recovery from IAV infection and the effect of IL-5 is independent of virus clearance

**Fig 1:**
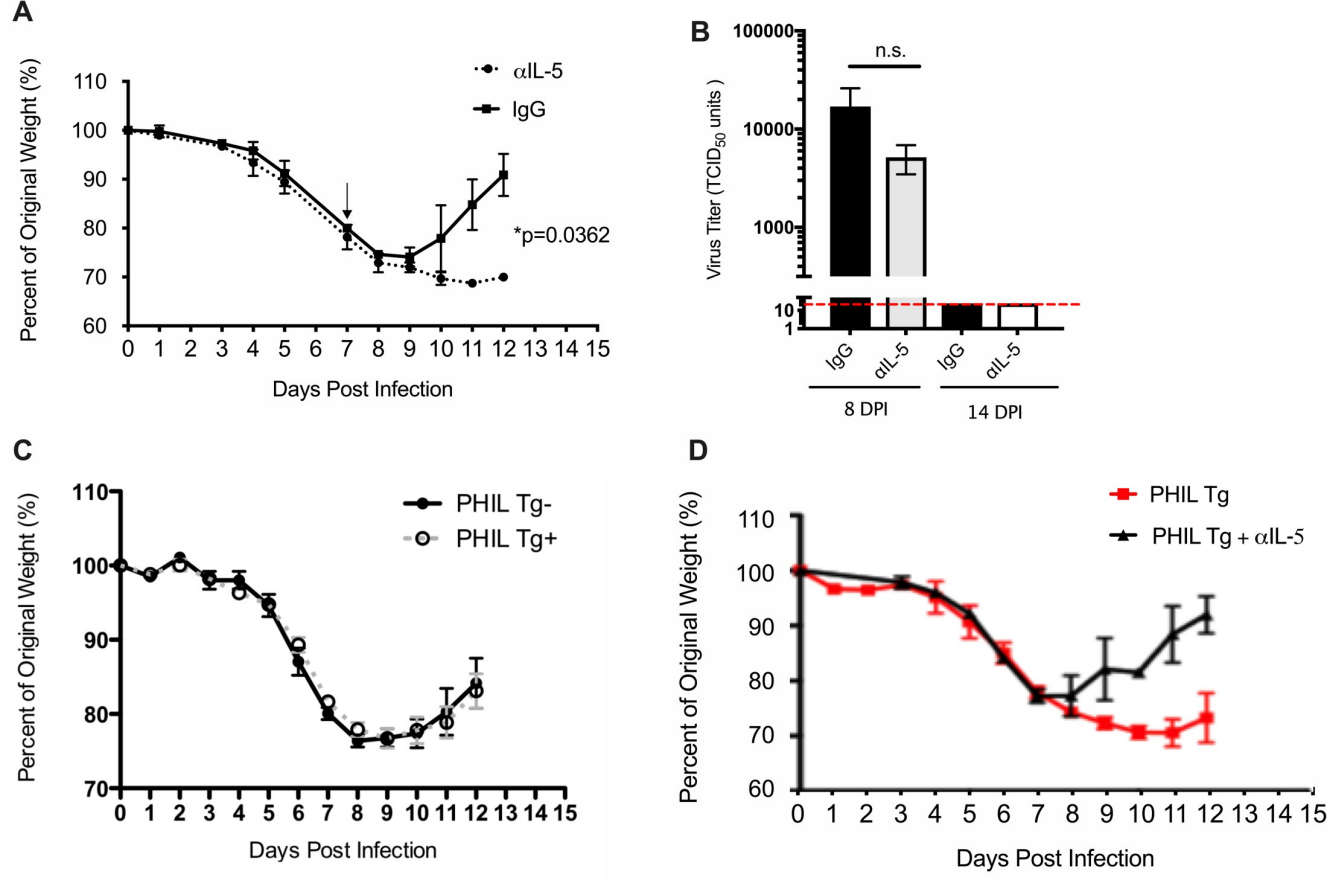
IL-5 is required for optimal recovery from IAV infection and is independent of virus clearance and eosinophils. (A) BALB/c mice were infected with 0.05 LD_50_ IAV and given 100 μg of αIL-5 i.p. daily beginning at 7 d.p.i. (denoted by arrow). (B) Virus titer from BAL fluid taken at indicated d.p.i. Red line denotes limit of detection. (C) PHIL mice on the B6 background were infected with 0.1 LD_50_ IAV and monitored for weight loss. (D) PHIL mice positive for DTα (i.e. eosinophil deficient; PHIL Tg+) were treated with αIL-5 or control IgG antibody and monitored for weight loss. Data are represented as mean +/- SEM (n = 3–21). Data are representative of at least two independent experiments. Student’s t-test was used to compare groups.

We have previously reported that ILC2 accumulate in the lungs of mice following experimental IAV infection and release large quantities of IL-5 into the BALF, with a parallel increase in eosinophilic inflammation in lungs as would be expected given the well-recognized role of IL-5 as an eosinophil chemoattractant and survival factor [16]. These data suggested that ILC2-derived IL-5 (and potentially eosinophils) might have a role in recovery from IAV infection. In order to better define the role of IL-5 specifically during the recovery phase of infection, we treated infected mice with a neutralizing IL-5 monoclonal antibody (αIL-5 mAb) daily, beginning at 7 d.p.i. as this was the time point of maximum IL-5 release into the BALF and precedes the time period during which the most significant eosinophil accumulation occurs (i.e. 8–16 d.p.i. [16]. Mice treated with αIL-5 mAb displayed a marked delay in recovery as measured by return to original weight (Fig 1A). Of note, there was no significant difference in virus titer between αIL-5-treated and IgG-control-treated IAV infected mice at 8 d.p.i. (i.e. one day following αIL-5 treatment) and infectious virions were undetectable by 14 d.p.i. in both groups (Fig 1B), suggesting that the protective effect of IL-5 was independent of virus clearance.

### Delayed recovery in αIL-5 treated mice is independent of eosinophils

Since αIL-5 treatment did not appear to affect viral clearance, we hypothesized that the delayed recovery observed in the αIL-5 treated mice possibly reflected a protective role for eosinophils in the resolution of infection and the recovery process. Indeed, there is evidence that eosinophils could play a protective role in modulating the severity of IAV infection [21]. To explore this possibility, we utilized the eosinophil-deficient PHIL mouse strain, to determine if the loss of eosinophils would influence the outcome of IAV infection. PHIL mice express the diphtheria toxin alpha chain (DTα) under the eosinophil-specific *Eosinophil peroxidase* promoter and thus are selectively deficient in eosinophils [22], which we confirmed via FACS analysis. Recovery from IAV infection of PHIL mice that were positive for the DTα transgene (PHIL Tg+) (i.e. eosinophil deficient mice) was comparable to that of littermate, wildtype controls (PHIL Tg-) (Fig 1C). Importantly, αIL-5 treatment of PHIL Tg+ mice resulted in delayed recovery (Fig 1D). These results suggested that IL-5 is acting *independently* of eosinophils during the recovery phase.

### Murine neutrophils express the IL-5 receptor

**Fig 2:**
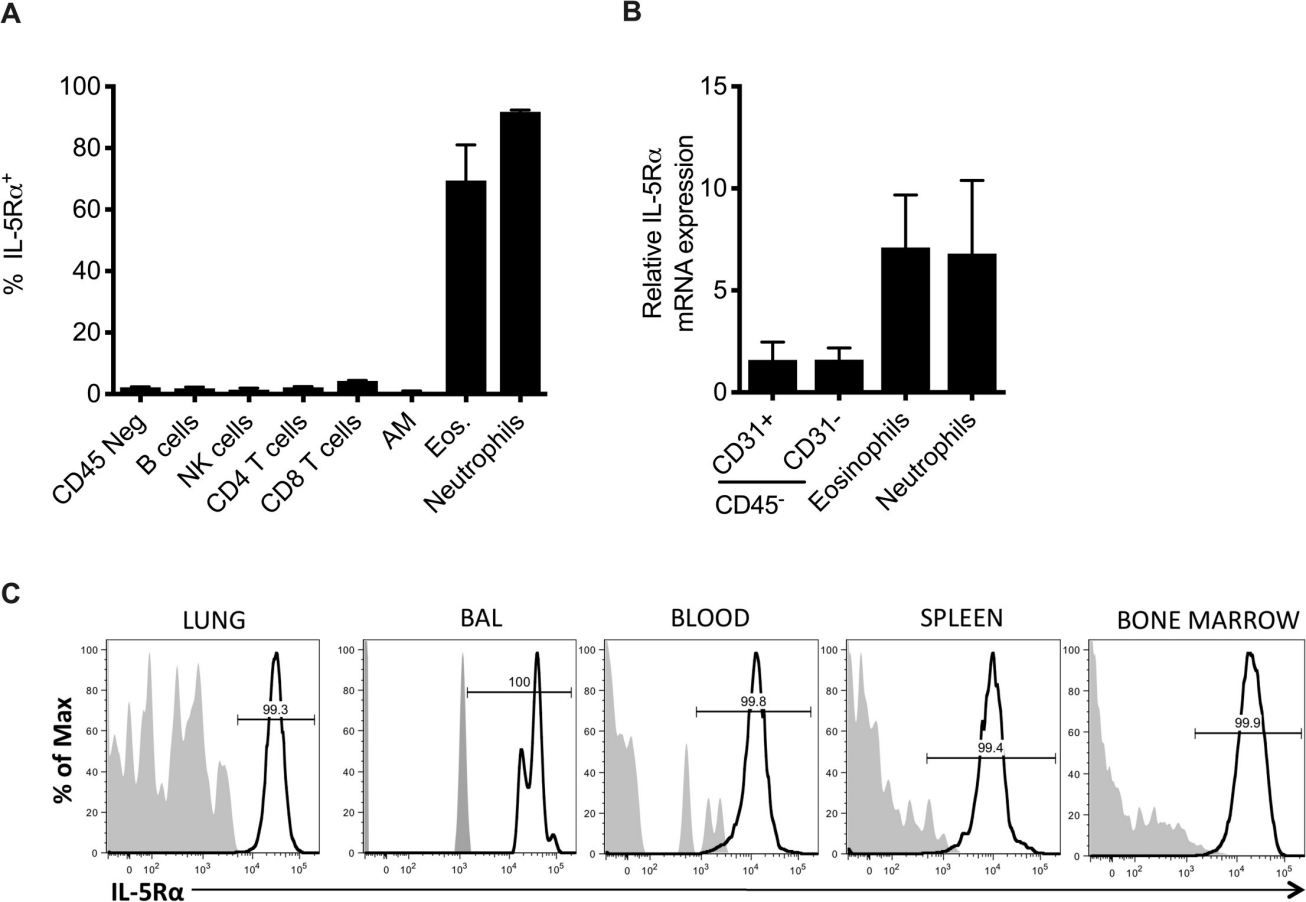
Neutrophils express the IL-5 receptor. (A) BALB/c mice were infected with 0.05 LD_50_ A/PR/8/34. Indicated cell populations from 10 d.p.i. lungs were interrogated for surface IL-5Rα expression. (B) Neutrophils (defined as CD45+CD11b+Ly6G+) were examined in indicated tissues in infected mice for IL-5Rα expression. Shaded histogram = isotype control. (C) Cell populations were FACS-sorted from the lung at 10 d.p.i. and analyzed for IL-5Rα gene expression by real time PCR. Data are represented as mean +/- SEM (n = 6–10). Data are representative of at least three independent experiments.

The surprising finding that IL-5 was acting independently of eosinophils to promote recovery from IAV infection raised the possibility that another cell type was responsive to IL-5 signaling and was capable of mediating pulmonary repair. We examined CD45+ and CD45- cell types in the lung for expression of the IL-5Rα (CD125) and found that in addition to eosinophils, surprisingly >95% of neutrophils also expressed IL-5Rα (Fig 2A) at 10 d.p.i. The expression of IL-5Rα was not unique to lung neutrophils as neutrophils analyzed in the BAL, blood, bone marrow and spleen were found to be virtually 100% positive (Fig 2B). Furthermore, FACS-sorted neutrophils from a 10 d.p.i. lung demonstrated equivalent IL-5Rα mRNA expression as eosinophils (Fig 2C). There was no significant difference in serum IL-5 levels between αLy6G-treated (i.e. neutrophil depleted) and IgG-control-treated IAV infected mice at 10 d.p.i. (S2 Fig).

This may be explained by the fact that the primary cellular source of IL-5 following IAV infection is ILC2 that are recruited into the lung, and therefore any significant serum IL-5 present is from diffusion into the circulation.

### Neutrophil respiratory burst is suppressed by IL-5

**Fig 3:**
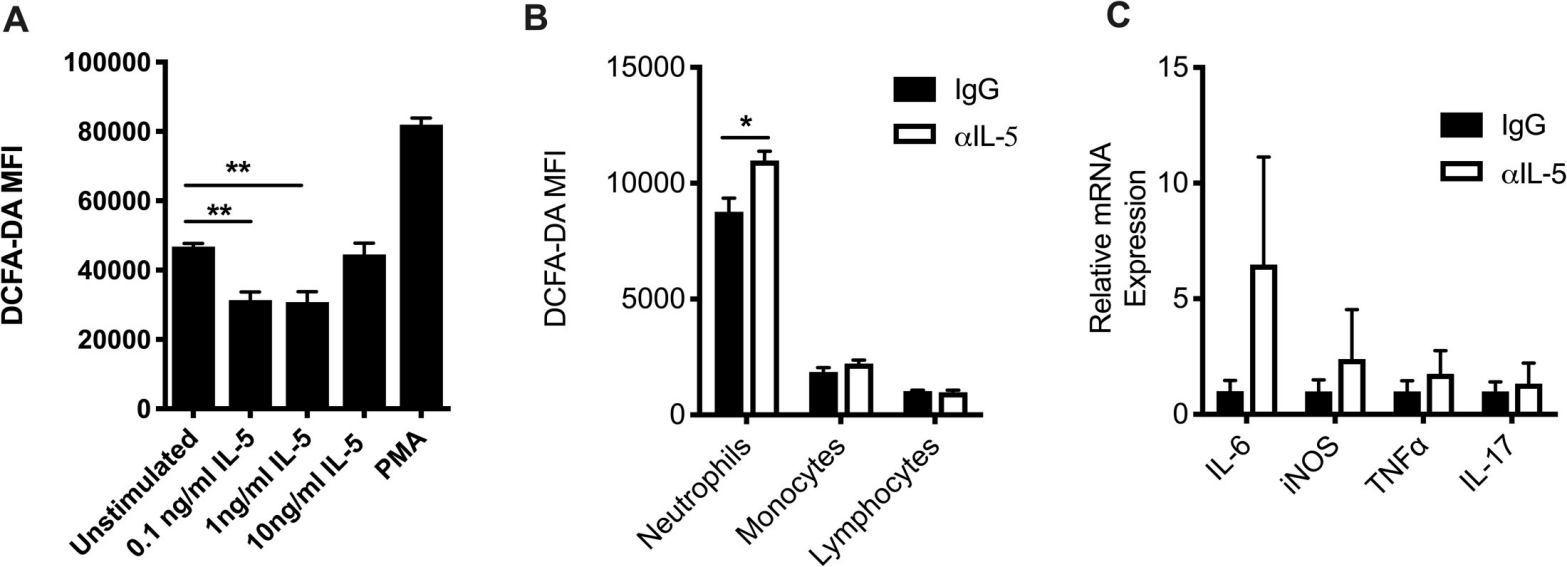
Neutrophils are responsive to IL-5 signaling. (A) Whole lung cell suspensions were kept in culture with indicated stimuli for 1 hour prior to addition of the ROS indicator dye DCFA-DA. Neutrophil DCFA-DA MFI was analyzed 30 minutes following the addition of DCFA-DA to whole lung cell suspensions. Data are represented as mean +/- SEM (n = 4 per group). Data are representative of four independent experiments. **p<0.01, ***p<0.001, ANOVA. (B) MFI of ROS indicator dye DCFA-DA in indicated populations from 12 d.p.i. lung. Lymphocytes were identified as CD11b- and low FSC/SSC; monocytes were identified as CD11b+Ly6G-. Data are represented as mean +/- SEM (n = 3–13). *p<0.05, Student t-test. (C) Neutrophils were FACS sorted from the lungs at 12 d.p.i. and analyzed for gene expression via real time PCR. Data are representative of at least two independent experiments.

In order to determine if the IL-5R expressed by neutrophils is functional, we treated infected lung-derived neutrophils with increasing concentrations of recombinant murine IL-5 (rmIL-5). Neutrophils treated with rmIL-5 had significantly less ROS production as measured by DCFA-DA indicator dye compared to unstimulated neutrophils (Fig 3A). The relatively high amount of ROS production in the unstimulated cells most likely reflects the elevated activation state of neutrophils in the recovering lungs *in vivo*. Whereas lower concentrations of rmIL-5 inhibited ROS production, neutrophils treated with 10ng/ml rmIL-5 showed no difference in ROS production compared to unstimulated cells, possibly due to down-regulation of the high affinity IL-5Rα.

The ability of neutrophil activity to be suppressed *in vitro* by IL-5 was surprising, and therefore we sought to determine if neutrophils have enhanced pro-inflammatory activity in the setting of IL-5 neutralization. Neutrophils from the lungs of 10 d.p.i. α-IL-5-treated mice produced significantly more ROS than 10 d.p.i. neutrophils from control IgG treated influenza infected animals (Fig 3B). Importantly, ROS production in monocytes or lymphocytes was comparable between αIL-5 and IgG treated mice, suggesting that IL-5 is acting directly on neutrophils. In support of IL-5 acting to suppress neutrophil effector activity, FACS-sorted neutrophils from 10 d.p.i. αIL-5 treated mice also had slightly higher gene expression for *inducible nitric oxide synthase (iNOS)*, an enzyme essential for the production of NO, a type of ROS. Furthermore, neutrophils from the αIL-5 treated mice had higher expression of IL-6, although there was a high degree of variability among different mice. Levels of TNFα and IL-17, other notable inflammatory neutrophil products, were comparable between αIL-5 and IgG treated mice (Fig 3C).

### Human neutrophils can express the IL-5 receptor

**Fig 4:**
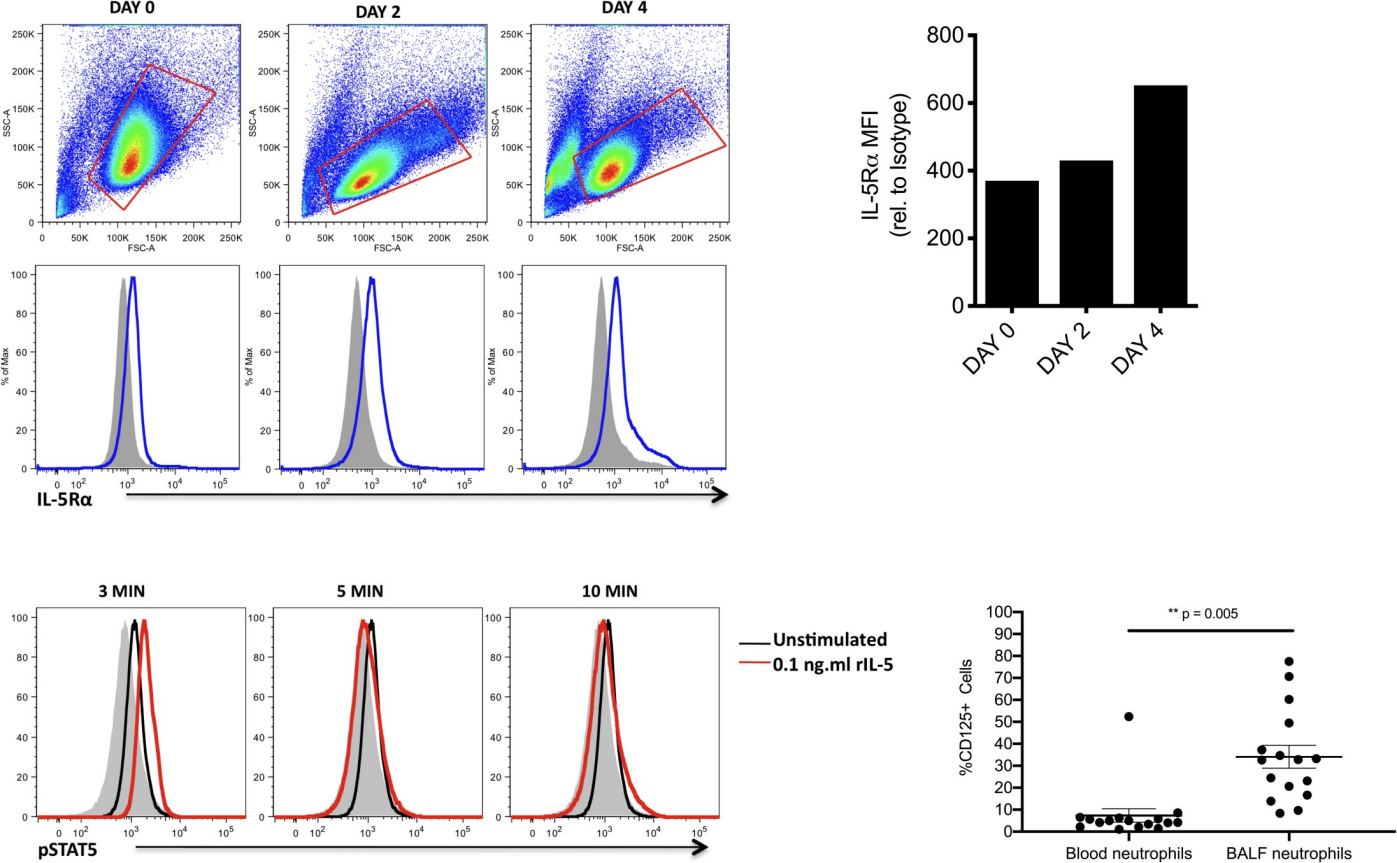
Human neutrophils can express the IL-5 receptor. (A) HL-60 cells were treated with 1.5% DMSO over the period of 4 days and analyzed for IL-5Rα (CD125) expression. Shaded histogram = isotype control. Data are representative of two independent experiments. (B) MFI levels of IL-5Rα on HL-60 cells relative to MFI of isotype control. (C) HL-60 cells treated with DMSO for 4 days were stimulated with 0.1ng/ml rIL-5 and analyzed for pSTAT5 at indicated times. (D) Neutrophils (defined as SSChigh, CD45+CD66b+Sig8-) were interrogated for surface IL-5Rα expression in paired BALF and blood samples (n = 16). p = 0.005 using Wilcoxon matched-pairs signed rank test.

To determine if IL-5Rα was also expressed on neutrophils in the humans, we next incubated HL-60 cells in the presence of DMSO to induce differentiation into human neutrophil-like cells [24]. This resulted in a steady increase of IL-5Rα expression out to day 4 (Fig 4A and 4B). Treatment of these cells with rhIL-5 also resulted in a transient increase of pSTAT5 following 3 minutes of stimulation, which was rapidly lost within 5 minutes post-treatment (Fig 4C), presumably due to phosphatase activity. This indicates that IL-5 is capable of inducing phosphorylation of STAT5 directly in human neutrophils.

To confirm these findings *ex vivo*, we next examined BALF neutrophils from 16 children undergoing clinically indicated bronchoscopy for persistent respiratory symptoms. Eleven of these children had moderate to severe, treatment-refractory asthma; one had cystic fibrosis; two had recurrent respiratory infections; one had tracheomalacia; and one had virus-induced wheeze. A comparison group of BALF from healthy control children was not available for analysis since bronchoscopies were only performed when clinically indicated. The age range of these patients was 0.7 to 15.8 years (median 5.3 years); four (25%) were black and 12 (75%) were white; and 14 (87.5%) were male while two (12.5%) were female. Fourteen of 16 were being treated with inhaled corticosteroids, 7 of 16 with long-acting beta-agonists, 7 of 16 with anti-leukotrienes and 2 of 16 with daily oral corticosteroids, while none were being treated with biologic therapies including anti-IL-5. Surface expression of IL-5Rα was detected on all BALF samples, with highly variable expression ranging from 8.4–77.5% positive cells (Fig 4D). In paired blood samples obtained from subjects undergoing bronchoscopy, IL-5Rα surface expression was again detected in all patients, but in 15 of 16 subjects, IL-5Rα was detected at a lower frequency in the blood than in the BALF (1.1–52.4% positive) (p = 0.005, Wilcoxon matched-pairs signed rank test).

## Discussion

We have previously demonstrated that IL-5 production by ILC2 during influenza infection leads to a mild pulmonary eosinophilia during the recovery stage [16]. The accumulation of eosinophils during the recovery phase of influenza infection seemed to suggest a potential role for IL-5 (and eosinophils) in the pulmonary repair process. However, while a role for IL-5 supporting recovery from infection was demonstrable, infection of PHIL mice, which are selectively deficient in eosinophils, surprisingly demonstrated that the recovery process in this model was independent of eosinophils (Fig 1C). One caveat to this model, however, is that the PHIL mice are deficient in eosinophils from birth, and the contribution of eosinophils (protective or deleterious) during the acute stage of infection or even prior to infection is not known. Importantly, αIL-5 treatment of PHIL Tg+ mice demonstrated that while eosinophils are not required, IL-5 is playing a positive role during the recovery phase of IAV infection (Fig 1D). The impact of IL-5 during the acute stage of IAV infection is unknown, but we suspect that the effects of IL-5 blockade would be minimal as myeloid lineage cells do not accumulate until the recovery phase.

In order to determine how IL-5 was mediating recovery independently of eosinophils, we analyzed all cell populations present in the lung for IL-5Rα expression and found that eosinophils and, unexpectedly, neutrophils were the major IL-5Rα+ cell types (Fig 2A). Analysis of IL-5Rα expression by neutrophils from IAV-infected mice showed that neutrophils express IL-5Rα in all tissues analyzed (i.e. blood, lung and bone marrow) (Fig 2B). The neutrophils were responsive to IL-5 signaling, with evidence of suppression of ROS production by IL-5 that was restored in the setting of IL-5 blockade (Fig 3). There was also evidence of STAT5 phosphorylation following IL-5 stimulation of differentiated HL-60 cells, a human neutrophil-like cell line, indicating that the IL-5Rα expressed by neutrophils is functional. These data suggest that during the recovery phase from IAV infection, ILC2 production of IL-5 both mediates the recruitment of eosinophils and dampens the inflammatory effector functions of neutrophils in the inflamed lung. Previous reports have suggested a protective role for eosinophils in anti-viral immunity and tissue repair [21, 26, 27]. The role of neutrophils in recovery from IAV infection has also been previously demonstrated (reviewed in [28]).

IL-5Rα expression on lung and blood neutrophils and macrophages was recently reported in humans in a sepsis model, where IL-5 was also shown to be protective in an eosinophil independent manner [29]. The authors reported that neutrophils do not express IL-5Rα in the naïve state, but rather, its expression was induced upon stimulation with LPS. Furthermore, calcium mobilization was detected in neutrophils and macrophages, and STAT1 phosphorylation was demonstrated in macrophages treated with rIL-5, indicating that the receptor was functional. Neutrophils isolated from the lungs in a mouse model of resuscitated hemorrhagic shock and tissue trauma have also been shown to express IL-5Rα expression [30]. These neutrophils were able to respond to IL-5 to produce their own IL-5 in a feed-forward mechanism, again indicating the the receptor is functional when expressed on neutrophils in an inflammatory state.

Our data provide evidence that neutrophils can play a role bridging type 2 and innate immunity, by providing the first evidence of IL-5Rα expression on neutrophils in the human airway in a type 2 inflammatory state (Fig 4). IL-5Rα expression has also been noted on neutrophils in the lungs of house dust mite sensitized mice [31] and in the lungs of horses with heaves, an allergic asthma-like condition [32]. In addition, other Th2 receptors, including the IL-9R (CD129) and VLA-4, have been described on neutrophils in human asthmatics as well as in murine models of viral asthma [33, 34]. There is also evidence of CD125 mRNA in neutrophils from the peripheral blood of healthy human donors [35]. Further studies with a larger sample size are needed to confirm the finding of IL-5Rα expression on human neutrophils in the airways of patients with type 2 inflammatory lung conditions. If confirmed, additional investigation will be required to understand the effects of IL-5-mediated signaling in neutrophils and define the relevant factors that lead to IL-5Rα expression on neutrophils.

This finding has important implications for treatment strategies for severe asthma. Current biologic treatments for severe asthma target a type 2-driven eosinophilic phenotype by blockade of IgE, IL-4Rα, IL-13, and, importantly, IL-5/IL-5Rα [27, 36]. These therapies have been designed to selectively target airway eosinophils, using peripheral blood eosinophil count as a surrogate marker of airway eosinophilia with the assumption that IL-5 mediates its effects via IL-5R found primarily on the surface of eosinophils. However, our data raise the possibility that at least some of the effects of these therapies are mediated by off-target effects on IL-5R+ neutrophils. It is increasingly recognized that many patients with severe asthma have neutrophilic or mixed granulocytic inflammation of the lower airways [25, 37–40] and thus, the potential to act on both eosinophils and neutrophils by targeting the IL-5/IL-5R axis has exciting treatment potential for both asthma and other chronic lung diseases such as COPD [41].

## Supporting information

S1 Fig Gating strategy to identify murine eosinophils and neutrophils. (TIF)

S2 Fig Serum IL-5 levels at 10 d.p.i. in neutrophil-depleted and wt mice. (TIF)

## Abbreviations

BALF: Bronchoalveolar lavage fluid
DCFA-DA: dichlorofluorescein diacetate
EID: egg infectious dose
d.p.i.: days post infection
IAV: Influenza A virus
ILC2: Group 2 innate lymphoid cell
i.n.: intranasally
i.p.: intraperitoneally
rm: recombinant murine
ROS: reactive oxygen species
