# Supplementary figures and images for: Expression of IL-5 receptor alpha by murine and human lung neutrophils
Source: PLoS One. 2019 Aug 15;14(8):e0221113. doi: 10.1371/journal.pone.0221113 (PMC6695150; doi:10.1371/journal.pone.0221113)

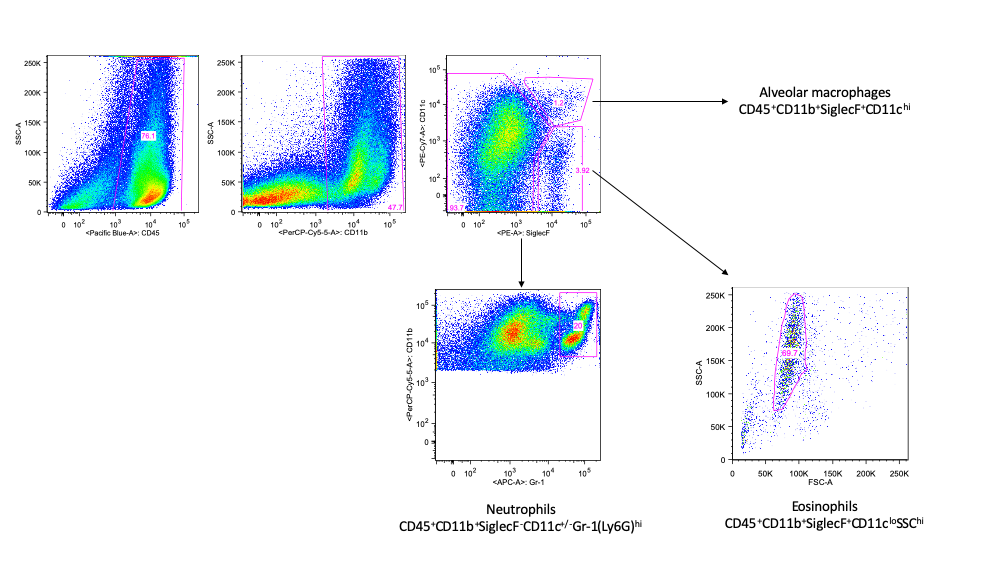

Supplement: S1 Fig — (TIF) [file pone.0221113.s001.tif]

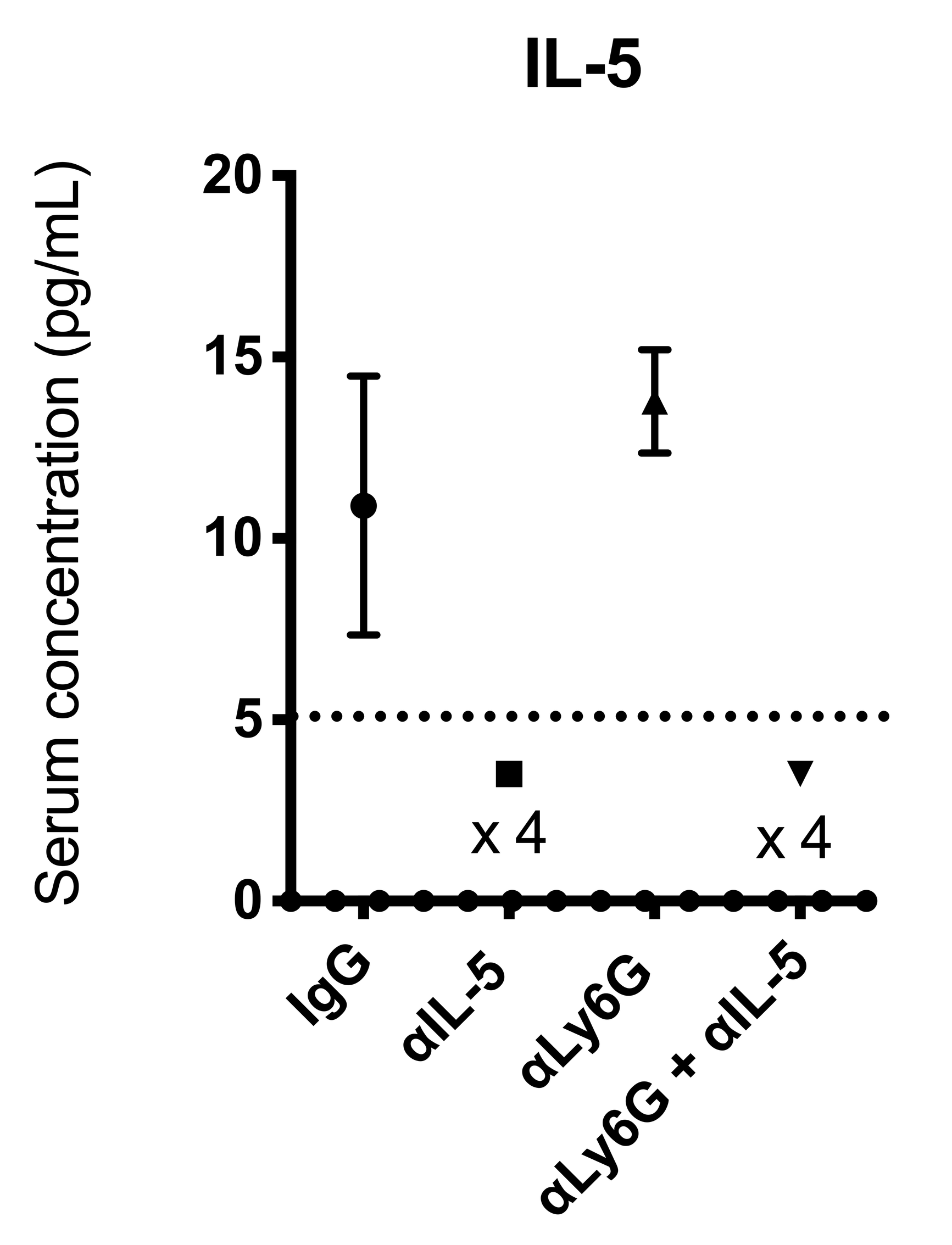

Supplement: S2 Fig — (TIF) [file pone.0221113.s002.tif]
